# Advances in Cryopreservation of Bull Sperm

**DOI:** 10.3389/fvets.2019.00268

**Published:** 2019-08-27

**Authors:** Muhammet Rasit Ugur, Amal Saber Abdelrahman, Holly C. Evans, Alicia A. Gilmore, Mustafa Hitit, Raden Iis Arifiantini, Bambang Purwantara, Abdullah Kaya, Erdogan Memili

**Affiliations:** ^1^Department of Animal and Dairy Sciences, Mississippi State University, Starkville, MS, United States; ^2^Department of Clinic, Reproduction and Pathology, Faculty of Veterinary Medicine, Bogor Agricultural University, Bogor, Indonesia; ^3^Department of Genetics, Faculty of Veterinary Medicine, Kastamonu University, Kastamonu, Turkey; ^4^Department of Reproduction and Artificial Insemination, Selcuk University, Konya, Turkey

**Keywords:** sperm, cryopreservation, extender development, fertility biomarkers, semen technology

## Abstract

Cryopreservation of semen and artificial insemination have an important, positive impact on cattle production, and product quality. Through the use of cryopreserved semen and artificial insemination, sperm from the best breeding bulls can be used to inseminate thousands of cows around the world. Although cryopreservation of bull sperm has advanced beyond that of other species, there are still major gaps in the knowledge and technology bases. Post-thaw viability of sperm is still low and differs significantly among the breeding bulls. These weaknesses are important because they are preventing advances both in fundamental science of mammalian gametes and reproductive biotechnology. Various extenders have been developed and supplemented with chemicals to reduce cryodamage or oxidative stress with varying levels of success. More detailed insights on sperm morphology and function have been uncovered through application of advanced tools in modern molecular and cell biology. This article provides a concise review of progress in the cryopreservation of bull sperm, advances in extender development, and frontiers using diverse techniques of the study of sperm viability. This scientific resource is important in animal biotechnology because with the advances in discovery of sperm fertility markers, there is an urgent need to improve post-thaw viability and fertility of sperm through enhanced cryopreservation for precision agriculture to produce food animals to ensure food security on the global scale.

## Introduction

There is an urgent need to improve the efficiency and sustainability of producing animals for food in the face of the ever-increasing world population. Increasing the fertility of livestock, especially cattle, around the world is important for overcoming this problem. Improved understanding of mechanisms and challenges of reproductive technologies are vital for improving the viability of the livestock industry. Among such reproductive technologies, Artificial Insemination (AI) is a significant technology that has been utilized to advance livestock farming, allowing for accelerated genetic progress and selection (1) where successful semen cryopreservation improves the efficiency and success rate of AI. Sperm cryopreservation procedures are not always efficient because a large number of sperm suffer physiological damage which leads to the loss of fertility following freezing and thawing (2).

The first reference to sperm cryopreservation dates back to the 1600s (3). Italian scientist Lazzaro Spallanzani successfully performed artificial insemination on bitches, which resulted in the live birth of three puppies after using cooled sperm in 1784 (4). Another 100 year later in 1899, Russian scientist Ilya Ivanovich Ivanoff developed practical methods of artificial insemination for farm animals. But there was little significant success or any widespread application until a discovery made by Phillips and Lardy in 1940 that poultry egg yolks can protect sperm from cold shock during cooling and maybe added to act as a cryoprotective agent (5). Salisbury et al. (6) further improved egg yolk usage as an extender by supplementing it with Na-citrate as a buffer to further preserve sperm at low temperature. The next major milestone in the field occurred when Polge et al. (7) uncovered the cryoprotective role of glycerol both at low temperatures and during the freezing process. Other advancements followed in the 1950s with the discovery of different extenders, packaging methods, and procedures which further improved the world-wide use of AI starting in the dairy industry (8).

Sperm cryopreservation is critical for livestock production because it enables and accelerate the spread of genetic diversity and it facilitates the distribution of genetically superior animals around the world. Due to the importance of cryobiology in reproductive technologies, new protocols are being developed and cryoprotectant agents tested for enhanced cryo-survival of sperm. Progress, however, has not yet reached a desired level because large portions of sperm die during the freezing-thawing processes (9). During these processes, sperm are faced with physiological and structural challenges due to changes in osmotic balance, oxidative stress, and the formation of intracellular ice crystals, hence, the need for supplements of antioxidants and cryoprotective agents (CPAs). In this paper, the challenges and current techniques to evaluate post-thaw viability of sperm will be discussed as well as the function of CPAs and antioxidants.

## Challenges In Sperm Cryopreservation

**Figure 1:**
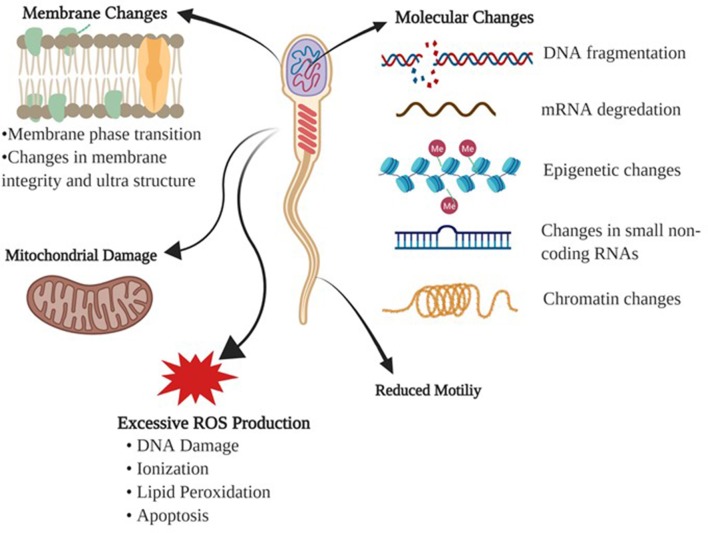
Detrimental effects of freezing-thawing on a sperm cell. Morphological and physiological effects of freezing and thawing processes on bull sperm are summarized.

Cryopreservation of sperm is a sequential process of reduction in temperature, dehydration of the cell, freezing, storage then thawing. Unlike other cells in the body, sperm cells should be less sensitive to their cryopreserving damage due to their low water content and high fluidity of the membranes. Despite this, cryopreservation is detrimental to sperm integrity due to alterations to the membrane structure-function and cell metabolism (10). Baust et al. (11) summarized the stressors influencing the cells during cooling and freezing stages as following: (1) During cooling, cells are exposed to many harmful effects including metabolic decoupling, ionic imbalance, activation of proteases, cellular acidosis, deprivation of energy, membrane phase transition, destabilization of the cytoskeleton, and production of free radicals or reactive oxygen species (ROS), (2) During process of freezing, sperm are predisposed to detrimental effects of ice crystal formation, hyper-osmolarity, alterations in the cell volume, and protein denaturation (Figure 1).

### Membrane Changes

The main cause of cellular injury in cryopreservation is the damage endured by the plasma membrane. Initially, it was assumed that cold shock was associated with the lipid composition of the membrane bilayer (12). When the temperature is lowered during the cooling process, restrictions of phospholipid lateral movement induce a change from liquid to gel phase causing the membrane to become more rigid and fragile. The phase changes involving the lipid membranes lead to lipid phase separation; thus, proteins are clustered irreversibly (13).

### Reactive Oxygen Species

During cryopreservation, any changes in mitochondrial membrane fluidity may result in the release of ROS and changes in the membrane potential (14). Hydrogen peroxide (H_2_O_2_), nitric oxide (NO), and superoxide anion (O_2_–) have positive effects on intracellular signaling, sperm capacitation, and acrosome reactions (15). Although at the appropriate levels of these molecules play a significant role in sperm physiology, namely capacitation and acrosome reaction, they are detrimental to sperm function at high concentrations due to toxicity. The exact mechanism of ROS generation and function have not been fully characterized in sperm (1). However, it is known that these molecules are products of incomplete reduction of molecular oxygen, and the toxicity is associated with protein inactivation due to ionization, lipid peroxidation, and DNA damage.

### Molecular Challenges

Identification of key molecular determinants of sperm freezability will aid in the development of better extenders and will provide insights to better predict fertility and sperm survival through cryopreservation. Such key determinants of freezability are generally evaluated by changes in parameters such as cell viability, motility and morphology, but current techniques have improved the aspects of novel assessments. Of these indicators of sperm quality, DNA integrity, and chromatin structure have been identified as the crucial factors regarding the ability of sperm to endure the cryopreservation process, and support embryo development (16). Freezing-thawing adversely affects DNA integrity making the DNA vulnerable and susceptible to molecular and epigenetic modifications, which affect the embryo development (17). This adverse process has been shown to induce chromatin destabilization which results in DNA fragmentation for boar and avian sperm (18, 19). DNA damage is likely related to several mechanisms which occur during cryopreservation; double strand breaks due to high levels of ROS production (20), impairment of DNA repair enzymes (21), and mechanical stress of genomic regions of the DNA molecule in which chromatin compaction is increased because of cell shrinkage (22). Apoptosis has been correlated with cryoinjury of sperm DNA and that excessive generation of ROS causes DNA damage (23). This can be different between fertile and sub-fertile bulls (24).

Factors including protamine, DNA methylation, and histone modifications take place in an epigenetic state and play critical roles in spermatogenesis (25, 26). Additionally, the epigenetic factors influence gene expression that is dynamically regulated during cryopreservation (27). Both mRNA and small non-coding RNA molecules are an important element of intercellular structure and research has shown that they play a role in transcriptional and post-transcriptional regulation of spermatogenesis (28, 29), while also being involved in reproductive physiology during the freezing-thawing protocols (30, 31). Sperm RNA quantity can be easily affected during freezing-thawing cycles and some degree of these RNAs remain stable in response to insult (25). Cryodamage can also cause degradation to mRNAs (32), and thus disrupt protein function and expression levels of fertility related proteins (33, 34).

Conventional methods, such as real-time reverse transcription polymerase chain reaction (qRT-PCR) and complementary DNA (cDNA) microarray techniques have been widely used to profile gene products in cryopreserved sperm (35). However, next-generation sequencing technology has paved the way to the era of transcriptome and the introduction of powerful and rapid new tools for classifying global transcripts of several species. A small number of studies have focused on the global transcriptome of cryopreserved sperm for a few animal species. In the bull, sperm transcripts are present (30) and it was demonstrated that freeze–thaw cycles can lead to changes in transcriptomic profiles between fresh and frozen thawed sperm (35). Also, cryopreserved sperm show an altered presence of non-coding RNAs including microRNA and piwi-interacting RNA (piRNA) (36). Most recently, it has been shown that non-coding RNAs have been involved in sperm cryoinjuries during cryopreservation (37–39) and are linked to apoptosis and metabolic activity pathway alterations. Although sperm are transcriptionally silent, the presence of RNA in sperm can provide effects of cryodamage both on sperm and embryo physiology. Recently, cryopreserved sperm has been demonstrated to influence transcriptomic profiles of embryos (40).

DNA methylation is part of the epigenetic mechanism and refers to the covalent addition of a methyl group to the DNA strand. This mechanism modulates gene expression in a variety of cells. Accurate DNA methylation in sperm is indispensable for early development and embryogenesis. The global level of DNA methylation is correlated with sperm parameters such as motility and concentration while chromatin fragmentation can adversely affect DNA methylation (41). However, aberrant DNA sperm methylation is linked to infertility in human and bovine (42). DNA methylation in sperm changes during freezing-thawing cycle that global methylation is increased after cryopreservation (43). This can also be supported with the regulation of epigenetic related genes which DNA methyl transferase (Dnmt3a and Dnmt3b) genes show *de novo* differential expression levels in cryopreservation (27). More specifically, a study in zebrafish corroborates that cryopreservation stimulates sperm hypermethylation in the promoters of important genes (44).

The functional chromatin integrity and packing of sperm genome are critical for the delivery of paternal DNA and epigenetic information to the oocyte (45). Several mechanical, physiological, and chemical factors can deteriorate chromatin integrity. There is increasing evidence suggesting that the sperm nucleus with altered chromatin structure provides additional information on cryodamage from freezing-thawing which cause alterations including denaturation (46). These changes influence the fertilizing capability of sperm without affecting functionality parameters (47). In most species and instances, low quality sperm have partly condensed chromatin which is susceptible to insult by polymerase and nucleases, resulting in DNA damage (48) and is associated with infertility in bull (49). Methods of freezing and the stage of cryopreservation can influence chromatin structure that it is mostly impaired at the thawing stage of cryopreservation (50, 51). Also, nuclear sperm alterations are attributed to cycles of freezing and thawing which subsequently link to DNA damage (46, 50).

## Molecular Markers Of Sperm Freezability

Seminal plasma and sperm proteins play important roles in sperm survival, fertilization, and energy metabolism (52). Recent studies showed that protein compositions and their expression levels in seminal plasma and sperm are associated with freezability differences among bulls. Some of the bovine seminal plasma proteins bind phospholipids of the sperm plasma membrane and hinder the movement of phospholipids. Expression levels of heat-shock protein (HSP90) were higher in semen with greater cryotolerance (34), and the levels of the HSP90 in bull sperm were significantly decreased in bull spermatozoa with lower cryotolerance (53). Holt et al. (54) claim that lower concentrations of heat shock protein A8 (HSPA8) in freezing media cause reduced post-thaw sperm viability, whereas higher concentrations improve plasma membrane integrity.

The cryopreservation process initiates carbonylation of bull sperm proteins. Mostek et al. (55) identified 11 proteins in bull semen (NADH dehydrogenase, ropporin-1, actin-related protein T2, outer dense fiber protein 2, glutathione S-transferase, triosephosphate isomerase, capping protein beta 3 isoform, actin-related protein M1, isocitrate dehydrogenase, cilia- and flagella-associated protein 161, phosphatidylethanolamine-binding protein 4) that they showed significant carbonylation levels during cryopreservation. Jobim et al. (56) found that presence of lipocalin-type prostaglandin D synthase (L-PGDS) is associated with poor freezability of bull sperm. The expression level of an acidic seminal fluid protein (aSFP) is higher in semen from high freezability sperm than that from low freezability. It assumed that aSFP plays a key role in protecting sperm from the damaging effects of oxidative stress by reducing lipid peroxidation (57).

## Extender Development

### Current State of the Art in Extenders for Bull Sperm

The cold shock endured during freezing and thawing reduces the quality of sperm. The extent of injuries from cold shock vary according to contents of extenders, cryoprotectants used, and species (58). A number of extenders have been developed to lessen cryodamage and improve post-thaw viability. Extenders based on 20% egg yolk are commonly used to cryopreserve livestock sperm of cattle, buffalo, and pigs (59). Although egg yolk is known to prevent cell damage during cryopreservation, the presence of substances in yolk granules including high-density lipoproteins (HDL) and minerals inhibit respiration of sperm cells and reduce their motility (60). However, the low density lipoproteins (LDL) of egg yolk protect sperm from damage by covering the sperm membrane during freezing and thawing (61). Although most extenders include egg yolk alone, some are supplemented with glycerol, and there are some concerns over biosecurity and the possibility that egg contents might alter sperm structure and physiology (62).

There have been efforts to develop commercial extenders with defined contents and those that are free of animal products. Recent studies performed by Murphy et al. (63) and Yodmingkwan et al. (64) revealed that plant-based extenders can be efficiently used as alternatives to animal-based extenders in frozen semen to avoid spread of diseases. However, there are conflicting results related to the efficacy of lecithin-based extender for semen freezing. Vidal et al. (65) claimed that there were no significant difference in post-thaw goat semen parameters between semen extended in Soy-lecithin based extender *vs*. Skim milk-based extender. Aires et al. (66) referred that the Andromed® extender containing soy-lecithin was better when compared to an egg yolk extender in cryopreservation of sperm from Holstein bulls. Additionally, Chelucci et al. (67) reported that soy-lecithin based extender preserves frozen goat semen better than that of an egg yolk based extender. However, according to Muiño et al. (68) a Biladyl® extender containing tris-egg yolk gave better results in terms of survival of sperm that those from Andromed® and Biociphos®. Other researchers claim that Tris-egg yolk-based extender is better in preserving frozen semen than plant-based extenders (69, 70). Moreover, studies on liposome based extenders have showed its effectiveness over both animal based and plant based extenders in preserving the frozen semen of buffalos (*Bubalus bubalis*) (71). There is a desperate need for more research and more extensive analyses of sperm as well as follow up studies on pregnancy rates and live births of offspring using sperm cryopreserved with different extenders.

### Cryoprotectant Supplementation of Extenders

Nonpenetrating and penetrating cryoprotectants are used to protect sperm cells from physical and chemical stressors caused by ice crystallization. While non-penetrating cryoprotectants such as polymers help with vitrification, penetrating cryoprotectants such as sugars help reduce toxicity. Cryoprotective agents cannot not prevent the changes in membrane phase but they can decrease the rate of dehydration during freezing which then lessen the formation of ice crystals in the cell (72).

Although glycerol, ethylene glycol, dimethyl sulfoxide, and 1,2-propanediol are all CPA, glycerol is the most commonly used in bovine sperm because it causes dehydration of cells by creating osmotic stimulation (73). Concentrations of CPAs differ among extenders; skim milk based extenders contain 8%, Tris eggyolk extenders contain 6–7% CPA. Therefore, the volume of intracellular water decreases and the chance of the ice formation reduces. However, glycerol may cause osmotic stress and toxicity (74, 75). In addition, sugars and polyols are used as cryoprotectants in semen processing. They create hydrogen bonds with membrane lipids; thus, lipids of sperm membrane are stabilized at low temperatures (2). Milk diluents are commonly used as CPA in bull semen. However, they have the disadvantage of decreasing the visibility of sperm cells under the microscope during sperm evaluation due to fat globules (76). Cryopreservation procedures cause significant losses of total lipids and phospholipids of sperm cells (77). Due to the importance of fatty acid composition on membrane fluidity, supplementations of fatty acids to the extender affect freezability. When the egg yolk-based extender was supplemented with docosahexaenoic acid, a major fatty acid in fish oil, sperm post-thaw viability has increased (78).

More recently, addition of 8% coconut oil as a source of lauric acid to egg yolk based-extender (79), 5 ng/ml α-linoleic acid to the BioXcell ® (80), and 20 ng/ml arachidonic acid to tris-citric acid extender (81) enhanced quality of sperm following cryopreservation. Additionally, Iodixanol is commonly used as a medium for density gradient centrifugation. Supplementation of sperm cells with Iodixanol (OptiPrep™) increases motility of buffalo sperm post-thaw (62). Mechanisms of Iodixanol actions are not clearly understood but it has been assumed that it protects sperm membrane through reducing ice crystal formation (82).

### Antioxidant Supplementation of Extenders

Antioxidants are molecules that inhibit the formation of ROS and lipid peroxidation. Superoxide dismutase, glutathione peroxidase, and catalase are the well-known antioxidants that are significant for sperm function because they protect sperm cells from oxidative stress (83). Glutathione (GSH) is a powerful antioxidant that protects bull sperm against free oxygen radicals and supplementation of buffalo semen with GSH increased motility, integrity of plasma membrane and cell viability (84). Another critical antioxidant for sperm integrity is Resveratrol which extinguishes superoxide, hydroxyl, and metal-induced radicals. Therefore, it protects sperm chromatin and membranes from ROS damage (85). Vitamin E also plays an important role on sperm membrane protection as an antioxidant. Supplementation of semen with vitamin E affects sperm motility, membrane integrity, and membrane potential positively (86). Endogenous antioxidants present in bovine semen are not sufficient to ensure sperm integrity against oxidative stress in cryopreservation. The supplementation of antioxidants is needed to improve the viability of post-thawed sperm cells. Bovine Serum Albumin (BSA) protects the sperm plasma membrane and acrosome, and physiology such as motility. Sariözkan et al. (87) found that the addition of BSA helped to maintain the cell morphology and acrosome integrity, and increased its catalase (CAT) activity. In addition, methionine is a precursor for glutathione which protects sperm from oxidative damage and is involved in detoxification of the cell. Moreover, the addition of methionine to semen helped to maintain normal sperm morphology. Furthermore, addition of carnitine and inositol to extenders has shown to have a protective influence on acrosome integrity as well as improved sperm motility and reduced DNA damage (88).

Plant-derived extracts are sources of natural antioxidants with lower cytotoxicity as compared to artificial antioxidants. Khan et al. (89) found that adding green tea extract at an inclusion level of 0.75% protected the plasma membrane and increased motility rates of cryopreserved spermatozoon. The addition of Spirulina maxima Extract (SME), a microalga, to extender has exhibited positive effects on post-thaw semen parameters including sperm motility and morphology, and marked reduction in ROS synthesis (90). Other natural compounds have been found to act as an antioxidant. Trehalose is a sugar that functions as an antioxidant and was shown to protect the structure of the sperm cell from oxidative and cold shock damage. The addition of 100 mM of trehalose into semen extender improved sperm post-thaw motility, integrity of the membrane, and the activities of CAT and GSH (91). Furthermore, supplementing semen extender with 2 μg/ml of selenium, a potent antioxidant, improved morphology, and integrity of cryopreserved sperm (92).

### Vitamins and Other Supplementations of Extenders

Vitamins, known for their antioxidant properties, along with other compounds may be utilized to combat cryo-damage and improve overall post-thaw quality of sperm. Vitamin C has been tested as an additive to extenders for the purpose of improving sperm quality after the harsh challenges that are brought upon the cells by cryopreservation. Vitamin C acts as an electron donator, to neutralize free radicals that are generated from normal metabolic activity in addition to environmental challenges. This ability to donate electrons allows for the reduction of oxidative stress from ascorbate free radicals (AFR). In a study performed by Mittal et al. (93) supplementation of 5 mM vitamin C to pooled bull ejaculates significantly improved seminal characteristics and significantly decreased the number of observed abnormal sperm as compared to the control group measurements. Vitamin C supplementation to sperm extenders has also been studied in buffalo bulls and has shown greater post-thaw motility and percent of intact plasma (94). Feeding animals with ascorbic acid has also shown significant increases in physical semen characteristics of ejaculate volume and sperm concentration, contributed to difference in scrotal circumference, reaction time, and testicular volume, while also improving sperm output characteristics such sperm motility, and total counts for Egyptian buffalo bulls (95).

Herbal extracts and supplements are another up and coming area of untapped potential for the animal reproduction industry. Silymarin is one such extract with potent antioxidant properties that comes from the milk thistle plant *Silybum marianum* (96). Little research has been conducted utilizing silymarin in cattle but recently in a study performed by El-Sheshtawy and El-Nattat (97) the supplementation of silymarin improved preservability of sperm in both chilled and frozen bull semen samples. Rosemary, *Rosmarinus officinalis*, a common household herb, has also been investigated as a potential cryoprotectant. In a study performed by Daghigh-Kia et al. (98), researchers supplemented bull sperm samples with rosemary extract, GSH, and a combination of the two to determine how the semen would be affected after being subjected to cryopreservation procedures. Results showed that the inclusion of the rosemary extract treatment and the combination treatment improved post-thaw characteristics of bull semen. Semen supplements discussed in that section have been summarized in Table 1.

**Table 1:** Extender development for sperm cryopreservation.

**Supplement**	**Functions/Effects**	**References**
**CRYOPROTECTANTS**		
Egg yolk	Low density lipoproteins (LDL) in egg yolk bind cell membrane and form an interfacial film during the freezing process	(99)
Milk	Protein fraction of skim milk protects sperm cells from cryo-injury	(1)
GlycerolEthylene glycolDimethyl sulfoxidePropylene Glycol	Responsible for membrane lipid and protein rearrangement Reduce intracellular ice formation by increasing dehydration at lower temperature	(73) (100)
Trehalose	Replace the bound water surrounding macromolecules and protectively hydrate those macromolecules by substituting for water	(101)
Polyols	Create hydrogen bonds with membrane lipids; thus, lipids of sperm membrane are stabilized at low temperatures	(2)
Fatty acids • Docosahexaenoic acid (Fish oil) • Lauric acid (Coconut oil) • α-linoleic acid • Palmitic acid • Oleic Acid	Increase post thaw viability, motility, and acrosome integrity by improving plasma membrane fluidity and integrity	(79–81)
Iodixanol	It assumed that protects sperm membrane through reducing ice crystal formation; thus, increases post-thaw sperm motility	(62) (82)
Butylated hydroxytoluene	Enhances motility, acrosomal integrity, and membrane integrity by increasing membrane fluidity and reducing activity of the lipid peroxyl radicals	(15, 102)
**ANTIOXIDANTS**		
Glutathione	Glutathione supplementation increase motility, plasma membrane integrity, and viability	(84)
Resveratrol	Extinguishes superoxide, hydroxyl, and metal-induced radicals. Therefore, it protects sperm chromatin and membranes from ROS damage	(85)
Vitamin E	Affects sperm motility, membrane integrity, and membrane potential positively	(86)
Bovine Serum Albumin	Helps to maintain the cell morphology and acrosome integrity, and to increase its catalase (CAT) activity	(87)
Methionine	Maintain normal sperm morphology	(88)
CarnitineInositol	Improve acrosome integrity, sperm motility, and reduce DNA damage	(88)
Spirulina Maxima Extract	Increase the motility and viability of sperm cells, and reduce ROS synthesis and protect DNA structure	(90)
Selenium	Improve morphology and integrity of cryopreserved sperm	(92)
**VITAMINS**		
Vitamin C	Vitamin C supplementation increase post-thaw motility and percent of intact plasma	(94)

## Techniques To Evaluate Sperm Quality

Comprehensive analyses of sperm by using integrated diverse methods are necessary to assess the cell morphology at the molecular and cellular levels that are linked to cell function. For examples, most relevant, advanced, standardized techniques should be applied correctly to capture sperm cell, genetic, functional, and epigenetic content. To improve cryopreservation, accurate predictor of sperm motility, viability, membrane functionality, mitochondrial activity, and apoptosis parameters should be assessed by contemporary techniques.

### Microscopy

*Light Microscopy* has been a commonly used tool to evaluate basic quality parameters of semen including sperm motility, morphology, membrane integrity, and concentration. *Fluorescent microscopy* has been an essential tool in biology and reproductive sciences, because of wide array of fluorochromes. The use of fluorescence labeling enables identification of sub-microscopic cellular components. Fluorescent microscopy has been extensively used to analyze sperm viability (103), the sperm membrane, acrosome, and chromatin. In this microscopy method, cellular components of sperm function are stained with fluorescent probes to examine the DNA, membranes, or lectins (104). Sperm viability assay can be analyzed by fluorescence microscopy using LIVE/DEAD commercial kits, which are DNA-binding fluorescent stains (SYBR-14) and membrane-permeant stain (PI), respectively. Acrosome integrity can be analyzed using the sperm acrosome molecular marker *Pisum sativum* agglutinin linked to fluorescein isothiocyanate (FITC-PSA). Terminal transferase dUTP nick-end-labeling (TUNEL) can also be used to evaluate apoptosis by flow cytometry and fluorescence microscopy.

*Laser Confocal fluorescence microscopy* is a technique that obtains three-dimensional (3D) optical resolution with depth of focus and provides protein distributions in cellular compartments. The advantages of confocal microscopy are that it recognizes fluorescence in individual cells, provides multispectral flexibility, and avoids out-of-focus suppression. In a confocal fluorescent microscope, excitation of the specimen beam by a laser is concentrated through the first pinhole aperture and then the emitted light is obtained and focused by the second pinhole and subsequently measured by a detector. Depending on the instrument type, confocal setup is required before each experiment; excitation laser is set considering maximal excitation and emission of each fluorochrome. To focus on sperm, the objective is set depending on sperm size, and parameters such as pinhole and gain voltage are adjusted for fluorochrome tested. For the acquisition of images of sperm, instrument acquisition parameters such as bit dept, thickness, and image format are adjusted for detection. Confocal microscopy is used to evaluate sperm characteristics such as the acrosome, chromatin, and membrane. More specifically, cytoskeletal proteins, such as, spectrin, tubulin and actin in the head of sperm can be examined by laser confocal fluorescence microscopy (105–107), likewise, expression of surface proteins in sperm cells can be evaluated (108). This microscopy allows observation of sperm movement (109), but lack of qualification and quantification of various characteristics, and provides accurate visualization of mitochondria; can be used to analyze mitochondria functionality at the single-cell level (110), while also can be adjusted for tracking of motion of sperms with active mitochondria (111). In addition, it can be applied to ascertain localization of lipid peroxidation (112) and ROS in sperm (113).

*Electron Microscopy (EM)* uses the electron as a tool to utilizes a beam of accelerated electrons to develop a specimen image (114). This technique provides higher magnification and resolution than light microscopy. In light microscopy, visible light is used to magnify the image of a specimen by using optical lenses that are the range of 10–1,000 times magnification. EM is performed in a vacuum and directly focuses an electron beam on the subject and images are magnified by the means of electromagnetic lenses. This microscopy technique has the advantage of using shorter wavelength of electrons at accelerating voltage. EM considerably expands our understanding of ultrastructure and morphological characteristics of sperm (115). The two most common electron microscopes are Transmission electron (TEM) and scanning electron (SEM). In these advanced microscopes, electromagnetic lenses are used to focus the electron beam on the image. Essentially, TEM sends off electrons via ultrathin sample to detector and generates two-dimensional image, while SEM scans the secondary electrons reflected from the specimen's surface and composition to create a three-dimensional image. SEM is used to examine the surface of sperm cell at low resolution with extensive magnification (116). SEM is advantageous when investigating adverse effects of cryopreservation on sperm morphological changes (117). TEM can be applicable to reproductive medicine and the investigation of structure and function of sperm (118). EM has beneficial uses in the diagnosis of sperm morphological defects (118).

*Holographic microscopy and Raman spectroscopy* have a holographic microscopy format where samples are visualized by laser light, and the obtained images are used to define the position, orientation, and the 3D structures of a microscopic sample. This technique provides label-free, no contact visualization, and high-resolution recording with numerical focusing which allows for 3D quantitative imaging of specimen and enables live cell applications on sperm morphology and motility (119–121). Holographic microscopy can be employed to evaluate morphology and integrity of bull sperm (122). Recently, computational, lens-free, and on-chip microscopy tools have been developed to track sperm heads and trajectories in second ranges for each frame (123). Human sperm structure can also be assessed by holographic microscopy (119). Recently, this high-throughput technique with developed image reconstruction was used to track sperm heads and tail in 3D locomotion (124). Raman spectroscopy is a useful technique that facilitates the study of biochemical changes of cellular components. The Raman spectroscopy technique is sensitive and non-destructive and relies on direct inelastic light scattering from a laser source in which frequency of photons is directed on a sample and scattered photon is detected as Raman effect. This frequency provides detailed information about identificated molecules from vibrational transitions with molecular interaction and composition, such as proteins and DNA in normal and abnormal human sperm (125, 126). Raman microspectroscopy has the capability of evaluation of chemical changes and molecular features of human and bovine sperm cells (127–129). Additionally, it offers an analysis of live sperm with physiological status (130). Raman spectroscopy can be combined with holographic microscopy to assess sperm quality with relation to morphological and biochemical properties (131, 132).

### Computer-Assisted Sperm Analysis (CASA)

The CASA system, first established in 1980, has evolved into an accurate computer-based technique and software which provides quantitative measurements to assess the sperm motility and kinematics objectively and precisely. This technique uses the principle of capturing continuous images of motile sperm from a microscopic field and converts images into video images with different acquisition rates (frames s-1, Hz). Captured images are scanned to be visualized through dark field or negative-high-phase contrast in order to track motion of each individual sperm considering intensity of frame in pixels and the head (133, 134). CASA provides motility parameters [progressive motility (%), total motility (%)] and kinematic characteristics for evaluation of sperm such as velocity, linearity, and lateral displacement which defines trajectory. This widely used measure of sperm movement includes velocities such as straight-line (VSL), curvilinear (VCL), average pathway (VAP), linearity of forward progression (LIN, ratio of VSL to VCL), and Amplitude of lateral head displacement (ALH). With high quality hardware and open-source software, current CASA systems are also more useful for the measurement of sperm morphometry (dimension) while also allowing for assessment of sperm viability, concentration, morphology, and degrees of DNA fragmentation (135, 136).

### Flow Cytometry

Flow cytometry (FC) is an outstanding system which has made it possible to analyze thousands of single cells in a short time. Flow cytometry permits analyses of large numbers of sperm cells as well as individual cells with physical characteristics of a single spermatozoon measured by a fluorescent compound. It is composed of fluidics, optics, and electronics systems, which uses the measurement of physical optics and chemical fluorescence characteristics of particles in a fluid when it is passes through a laser source (137).

In brief, this technique requires a small amount of sperm cell suspension and particle samples labeled with fluorescent markers in suspension which can be injected into a flow cell in the instrument. Subsequently, the fluorescence is absorbed and wavelengths of fluorescence from particle emission are detected by two optic lenses, generating measurement of fluorescent bands. During data collection, non-sperm scatter is gated out, considering characteristics of spermatozoon and fluorescence is subtracted from the total fluorescent intensity. Two types of flow cytometry systems are available, one of which having sorting capabilities (fluorescence activated flow cytometry-FACS) allows physically separation and purification of cells (138, 139). The other is non-sorting, which measures fluorescent emission in a highly repeatable, accurate, and sensitive manner. It generates high-throughput data on subpopulations while also capturing the measurements of heterogenous populations such as sperm. The structure of multiple organelles of sperm can be simultaneously evaluated using flow cytometry:

*Cell viability analysis* helps identify viable and non-viable sperm which are associated with the molecular anatomy and physiology of the membranes. More specifically, during the cryopreservation process, changes in temperature and osmotic stress impair sperm viability because of injury to the plasma membrane (140). This method uses probes such as ethidium homodimer (EH) (141), propidium iodide (PI) (142), Yo-Pro-1 (143), and bizbenzimidazole Hoechst 33258 (144) dyes, alone, or in combination with other dyes, to excite lasers (145). Propidium iodide (PI) is excited with the 488-nm laser and able to penetrate the non-viable sperm through broken plasmalemma, then emit red fluorescence upon binding to nucleic acids (146). SYBR-14, viability probe, emits green fluorescence from nuclei upon entering active cells (147), and can be combined with PI. This staining technique can be modified with other stain combinations to assess acrosomal integrity or mitochondrial function (148). SYBR14-PI staining has been employed to evaluate the effects of cryopreservation on sperm viability in many species, such as bee (149), stallion (150), bovine (151, 152), and fish (153, 154). Yo-Pro-1 is a green cyanine probe which reaches emission at 509 nm and can be applied to study membrane permeability (155). Yo-Pro-1 is combined with a membrane permeable dye ethidium homodimer and carboxyseminaphthorhodal fluor-1 (SNARF-1) to assess membrane stability in cryopreserved sperm (86, 156). Also, a combination of Yo-Pro-1 and PI can be better practiced than SYBR-14/PI to detect early phase damages in the membrane (157). Fluorescent probe Hoechst 33258 requires the ultraviolet laser to excite at 352 nm and emits blue fluorescence at 461 nm when bound to the nucleic acid. Hoechst 33258 can be used to determine viable and non-viable sperm (158) and it also provides an option to be combined with other probes leaving the green-red detection available. Hoechst 33342 (H-42) is another cell-permeant nuclear dye that is excited by an ultraviolet laser at 350 nm and emits blue fluorescence at 461 nm after binding to DNA. Recently, this dye has been used with ethidium homodimer to differentiate live and dead sperm cells (159).

Lately, fixable viability dyes relying on reaction of fluorescence with cytoplasmic amines have become available to detect live and dead cells and applicable for multicolor experiments. Fixable dyes cannot pass through an intact live cell membrane, resulting in a weak staining. However, they can stain amines in the cytoplasm of damaged cells. Zombie Green™, a fixable dye, is excited at 488 nm with a blue laser has a maximum emission at 515 nm. This dye has been tested for evaluation of sperm viability and mitochondrial membrane potential (MMP) (150).

*Acrosome integrity*, an indication of intactness, is essential for fertilization and the subsequent penetration of sperm into the zona pellucida. Acrosome integrity assays have been widely analyzed by a number of methods such as phase contrast, fluorescence with probes, and electron microscopy (104). Instead, flow cytometry requires labeled acrosome with lectin probes conjugated with fluorochrome fluorescent isothiocyanate (FITC). For this purpose, due to specificity, *Arachis hypogaea* (peanut) agglutinin (PNA) and *Pisum sativum* (pea) agglutinin (PSA) are the most commonly used lectins (160). In damaged acrosomes, while PSA lectin recognizes α-D- glucosyl and α-D-mannosyl residues in acrosome (161) and stains acrosomal matrix, PNA binds to the outer acrosomal membrane (162, 163).

PNA label in damaged or reacted spermatozoa emits green fluorescence, but intact acrosomes cannot yield fluorescence (148). The integrity of plasma membrane as well as the acrosomal integrity are measured at the same time using the combination of FITC-PSA and PI which has been used in dog and cryopreserved bovine sperm (86, 148). PNA-FITC staining can be more precise in the detection of acrosome than PSA-FITC (164). Also, combination of FITC-PNA/PI staining is useful for semen quality, allowing for the assessment of viability, and acrosomal integrity with analysis of live/dead and intact/damaged ratio (165, 166) and evaluation of effects of cryopreservation on acrosomal status (167).

*Mitochondrial activity* is an indicator of sperm physiology has been analyzed using fluorochromes with flow cytometry to elucidate mitochondrial function in the sperm (168, 169). The 3,3′- Dihexyloxacarbocyanine iodide-484/501nm [DiOC6(3)], green fluorescence dye, has been previously employed to analyze semen (170). However, this dye tends to stain other organelles, such as Golgi apparatus, when used at higher concentrations and can be non-specific in determining membrane potential (171). Rhodamine 123 (R123), a green-fluorescent dye that was used to evaluate MMP, has been replaced by improved dyes such as MitoTracker or JC-1 dyes (103). R123 cannot differentiate high and low MMP because of high mitochondrial respiratory rates and can lose signals out of sperm cells when the MMP is weak (171, 172). MMP can be analyzed by using commercial dyes such as MitoTracker which works by permeating the cell and accumulating in the mitochondria. When it is bound to mitochondria, mitochondria steadily emit fluorescence even after the cell dies, thus allowing multicolor labeling in sperm (47, 173). MitoTracker dyes show a broad range of fluorescence (red, green, orange) and accumulate in active mitochondria after spreading across the plasma membrane. Of these, Carbocyanine-based MitoTracker® Probes, such as MitoTracker Green FM and MitoTracker Red FM, act as a marker for live cells dependent on MMP but cannot be retained after fixation. However, Rosamine-based MitoTracker® Probes (MitoTracker Orange CM-H_2_TMRos, MitoTracker Orange CMTMRos, MitoTracker® Red CMXRos, MitoTrackerR Red CM-H_2_XRos) are well-retained after aldehyde fixation. The Rosamine family of reduced MitoTracker dyes, MitoTracker Red CM-H_2_XRos and MitoTracker Orange CM-H_2_TMRos, do not emit fluorescence until oxidative respiration occurred and can be used for estimation of oxidation status in sperm cells. These probes have been utilized to assess sperm, specifically MitoTracker Deep Red and MitoTracker Green, have been used to determine MMP (150, 159, 174, 175). The 5,5′,6,6′-tetrachloro-1,1′,3,3′-tetraethylbenzimidazolylcarbocyanine iodide (JC-1), another mitochondrial dye, is more specific to MMP (138, 176), and enables the differentiation between low and high MMP with dual fluorescence shifting from green to orange. In inactive mitochondria, forming monomers emits green fluorescence (525–530 nm wavelength) after being excited with a blue laser at 488 nm when MMP is low. However, inactive mitochondria with high MMP, form J-aggregates reach maximum spectra at 590 nm and emit orange fluorescence once excited with yellow (561 nm) laser. JC-1 has been used to evaluate semen quality (177), and to show differences in the sperm mitochondrial function (103, 178).

### Oxidative Stress Analysis

Oxidative stress has detrimental effects on sperm by deteriorating fertilizing ability. This is caused by production of ROS including radicals such as hydroxyl radical (OH), superoxide anion (O2) and non-radical hydrogen peroxide (H_2_O_2_) (179). During cryopreservation and thawing, sperm cells undergo cold shock which then leads to excessive ROS and lipid peroxidation (180, 181). ROS and oxidative species can be detected with better accuracy and reproducibility using flow cytometry as compared to other approaches. The 2′, 7′-dichlorodihydrofluorescein diacetate (H_2_DCFDA) is commonly used as ROS indicator to measure intercellular H_2_O_2_. Nonfluorescent H_2_DCFDA penetrates into the cell membrane and becomes stable in the intercellular once cleaved by intracellular esterases. Upon oxidation, it emits green fluorescence at ~517–527 nm by conversion to fluorescent 2′,7′-dichlorofluorescein (DCF) form (182, 183). Dihydroethidium (hydroethidine) is a specific ROS indicator probe that can be employed to detect superoxide production (184). The reduced form is oxidized by superoxide and emits red fluorescence at 610 nm after intercalating into DNA (185). This probe can be combined with viability markers to better determine ROS generation in live cells (186) and can be applied to study intracellular ROS in sperm (151, 187, 188). The 5-(and 6) -chloromethyl-20, 70-dichlorohydrofluorescein diacetate (CM-H_2_DCFDA), an oxidative stress probe, shows better retention than H_2_DCFDA and measures hydrogen peroxide in intact cells. Upon moving into the cell, CM-H_2_DCFDA diffuses into the plasma membrane, and acetates are cleaved by cellular esterases and thiol-reactive chloromethyl group to form 20,70-dichlorodihydrofluorescein (H_2_DCF). Then, oxidation of H_2_DCF into DCF by H_2_O_2_ emits fluorescent at 525 nm once excited at 495 nm (189). This probe is also convenient for the assessment of oxidative stress in bull sperm (190). MitoSOX Red is a probe developed to quantify selectively cellular and mitochondrial superoxide productions in the mitochondria (191). MitoSOX Red reagent, oxidized by superoxide, and fluoresce red at 580 nm, can be successfully used in human and bovine sperm (140, 192, 193).

*Sperm chromatin structure* reflects the capability of sperm to fertilize the egg and is measurement of the sperm quality. The Sperm Chromatin Structure Assay (SCSA) relies on extend of DNA denaturation which then sperm samples are mixed with an acridine orange (AO), resulting in metachromatic shift from green fluorescence to red fluorescence (194). In this assay, when AO intercalates with double-stranded DNA (dsDNA) it yields green, but it yields red when it intercalates with single-stranded DNA (ssDNA). Determining the ratio from the mixture of green and red fluorescence, for each spermatozoon, by a 488-nm laser by flow cytometry demonstrates the status of DNA fragmentation (DNA fragmentation index) and the chromatin structure. Also, terminal transferase dUTP nick-end-labeling (TUNEL) can also be employed to evaluate sperm DNA fragmentation by flow cytometry. This assay requires the enzyme, terminal deoxynucleotidyl transferase to catalyze the ration where deoxyuridine triphosphate nucleotides are incorporated into DNA breaks at their 3′-hydroxyl ends. TUNEL and SCSA accompanied with flow cytometry have compatibility (195, 196) and produces accurate information related to sperm fragmentation.

*Sex sorting* is a practical application of flow cytometry which requires applicable protocols and high-speed cytometers. The main principle of this techniques is to determine the DNA content of individual sperms. In this technique, Hoechst 33342 fluorophore is incubated with sperm; it enters the cell and binds to DNA. Because the X-chromosome has a lot more DNA than the Y-chromosome, X- and Y-bearing sperm are separated using flow cytometry (197). During flow through in the stream, each spermatozoon is enclosed in droplets which are subsequently captured by fluoresce detector. A fluorescence signal from X or Y chromosomes is detected, and positive or negative charge is then assigned to droplets. As they pass through the oppositely charged plate, they are separated into either X or Y tubes consistent with their DNA contents (198, 199).

There is a need to develop a more comprehensive methodology and novel techniques for assessment of the quality and viability of sperm should be developed or combine new techniques including bioinformatics or mathematical biology with the current techniques to study post-thaw viability. Exploring important aspects of sperm cryobiology, for instance, functional genomics (transcriptomics, proteomics, lipidomics, and metabolomics) and epigenomics (DNA methylation and chromatin dynamics), can have significant positive impact on AI protocols. Novel biomarkers (proteins, small non-coding RNAs such as microRNAs, lipids and small molecules, or epigenomic markers) can be used to better understand spermatogenesis, sperm quality, predict male fertility, and develop better extenders.

## Author Contributions

MU, RA, BP, AK, and EM conceptualized the study. All of the authors helped to develop the manuscript.

### Conflict of Interest Statement

The authors declare that the research was conducted in the absence of any commercial or financial relationships that could be construed as a potential conflict of interest.
